# Variation of volatile organic compound levels within ambient room air and its impact upon the standardisation of breath sampling

**DOI:** 10.1038/s41598-022-20365-7

**Published:** 2022-09-23

**Authors:** Michael Jonathan Hewitt, Ilaria Belluomo, Simone Zuffa, Piers R Boshier, Antonis Myridakis

**Affiliations:** 1 Department of Surgery and Cancer, Imperial College London, London, UK; 2 Department of Metabolism, Digestion and Reproduction, Imperial College London, London, UK

**Keywords:** Biomarkers, Molecular medicine, Mass spectrometry, Metabolomics

## Abstract

The interest around analysis of volatile organic compounds (VOCs) within breath has increased in the last two decades. Uncertainty remains around standardisation of sampling and whether VOCs within room air can influence breath VOC profiles. To assess the abundance of VOCs within room air in common breath sampling locations within a hospital setting and whether this influences the composition of breath. A secondary objective is to investigate diurnal variation in room air VOCs. Room air was collected using a sampling pump and thermal desorption (TD) tubes in the morning and afternoon from five locations. Breath samples were collected in the morning only. TD tubes were analysed using gas chromatography coupled with time-of-flight mass spectrometry (GC-TOF-MS). A total of 113 VOCs were identified from the collected samples. Multivariate analysis demonstrated clear separation between breath and room air. Room air composition changed throughout the day and different locations were characterized by specific VOCs, which were not influencing breath profiles. Breath did not demonstrate separation based on location, suggesting that sampling can be performed across different locations without affecting results.

## Introduction

Volatile Organic Compounds (VOCs) are carbon-based compounds that are gaseous at room temperature and are the end products of many endogenous and exogenous processes^1^. VOCs have been of interest to researchers for several decades for their potential role as non-invasive biomarkers of human diseases. However, there remains ongoing uncertainty regarding standardisation of both the collection and the analysis of breath samples.

One crucial area of interest for breath analysis standardisation is the potential effect of background VOCs within the ambient room air^2^. Previous studies have suggested that background VOCs within the ambient room air influence the levels of VOCs detected within exhaled breath^3^. A study by Boshier et al*.* in 2010 utilising selected ion flow tube mass spectrometry (SIFT-MS) examined the levels of seven VOCs in three clinical environments. Differing ambient VOCs levels were identified across the three areas which in turn raised suggestions about the ability of VOCs of high prevalence in room air to be utilised as disease biomarkers^4^. In 2013, Trefz et al*.* also monitored the ambient room air of an operating theatre over the course of a working day alongside breath samples from hospital staff. They found levels of exogenous compounds such as sevoflurane had increased in both ambient room air and breath by the end of the working day^5^ raising questions as to when and where sampling of patients for breath analysis should be performed to minimise such confounding factors. This was correlated by a study by Castellanos et al. in 2016 who identified sevoflurane in the breath of hospital workers but not in that of workers outside of the hospital^6^. In 2018, Markar et al*.* attempted to demonstrate the impact of variation in room air composition on breath analysis as part of their study to assess the diagnostic capability of exhaled breath for oesophagogastric cancer^7^. They utilised steel breath bags and SIFT-MS for their sampling process and identified eight VOCs within room air that differed significantly across sampling locations. These VOCs however were not included within their final diagnostic model of breath VOCs and thus their impact was negated. In 2021, Salman et al*.* performed a study monitoring the VOC levels across three hospital locations over 27 months. They identified seventeen VOCs that acted as seasonal differentiators and proposed a cut off level of exhaled VOC concentrations above 3 µg/m^3^ as being unlikely to be secondary to background VOC contamination^8^.

Aside from setting a cut off level or direct exclusion of exogenous compounds, alternative methods to negate this background variation include collecting paired samples of room air at the same time as breath sampling so that the level of any VOCs present in high concentrations in the inhaled room air can be subtracted from levels found in the exhaled breath^9^ providing an “alveolar gradient”. A positive gradient is thus suggestive of an endogenous compound^10^. Another approach is to have participants inhale “scrubbed” air that is theoretically free from contaminant VOCs^11^. However, this is onerous, time consuming and the equipment itself can generate additional contaminant VOCs. A study by Maurer et al. in 2014 had participants inhale synthetic air which reduced the intensity of 39 VOCs, but increased intensity of 29 VOCs compared to inhaling ambient room air^12^. The use of synthetic/scrubbed air also significantly limits the portability of equipment for breath sampling.

It is also anticipated that the levels of VOCs within ambient air would alter throughout the day which could further impact upon standardisation and accuracy of breath sampling.

Advances in mass spectrometry techniques including the coupling of thermal desorption with gas chromatography and time-of-flight mass spectrometry (GC-TOF-MS) also provides a more robust and powerful VOC profiling approach, enabling the concurrent detection of hundreds of VOCs and consequently, a more in-depth analysis of room air. This provides the opportunity to present a more detailed characterisation of the composition of ambient room air and the variation across location and time with a larger number of samples.

The primary aim of this study is to determine the varying abundance of VOCs within ambient room air in common sampling locations within a hospital setting and how it potentially impacts exhaled breath sampling. Secondary aims were to determine if there is a significant diurnal or locational variation in VOC profiles in ambient room air.

## Results

### Breath and room air have distinct VOCs profiles

**Figure 1:**
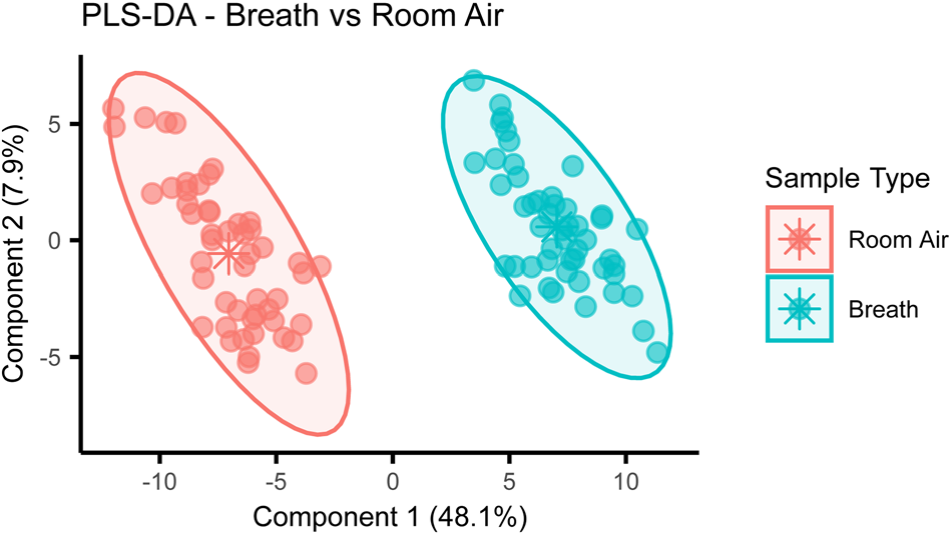
Breath and room air present distinct VOCs profiles. Supervised analysis with PLS-DA showed a clear separation between breath and room air VOCs profiles collected during the morning (R^2^Y = 0.97, Q^2^Y = 0.96, p < 0.001). Repeated measures were collapsed to the mean before model construction. Ellipses show 95% confidence intervals and asterisks group centroids.

Breath samples were collected in the morning alongside matching room air samples at five different locations and analysed by GC-TOF-MS. A total of 113 VOCs were detected and extracted from the chromatograms. Repeated measures were collapsed to the mean before performing principal component analysis (PCA) on the extracted and normalised peak areas to identify and remove outliers. Supervised analysis through partial least squares—discriminant analysis (PLS-DA) was then able to show a clear separation between breath and room air samples (R^2^Y = 0.97, Q^2^Y = 0.96, p < 0.001) (Fig. 1). Group separation was driven by 62 different VOCs, with a variable importance projection (VIP) score > 1. A complete list of the VOCs characterizing each sample type and their respective VIP scores can be found in Supplementary Table 1.

### Diurnal variation in room air VOCs levels

**Figure 2:**
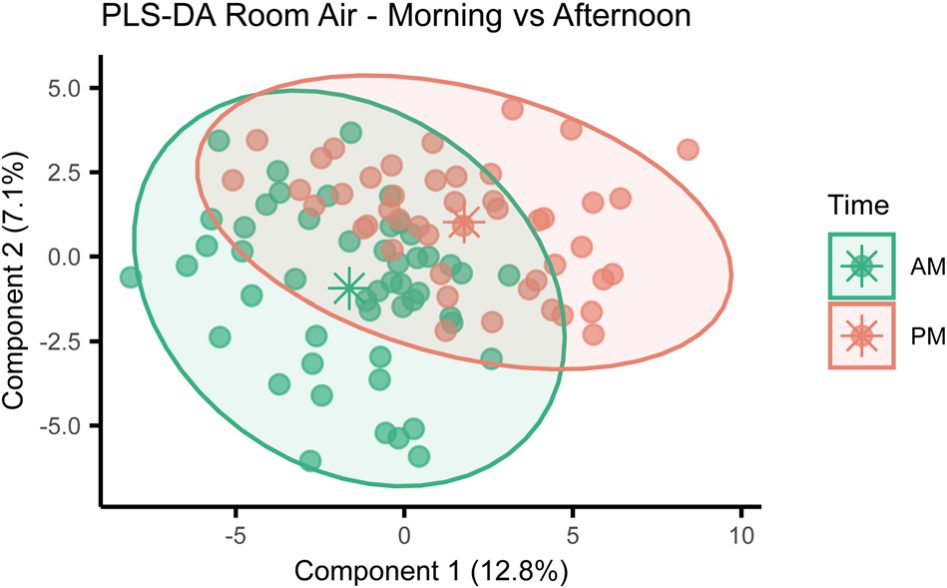
Room air VOC profiles change during the day. Supervised analysis with PLS-DA showed separation between room air samples collected during the morning or during the afternoon (R^2^Y = 0.46, Q^2^Y = 0.22, p < 0.001). Ellipses show 95% confidence intervals and asterisks group centroids.

Differences in room air VOC profiles between morning and afternoon were investigated using PLS-DA. The model identified significant separation between the two timepoints (R^2^Y = 0.46, Q^2^Y = 0.22, p < 0.001) (Fig. 2). This was driven by 47 VOCs with a VIP score > 1. VOCs with the highest VIP score characterizing morning samples included multiple branched alkanes, oxalic acid and hexacosane, while afternoon samples presented more 1-propanol, phenol, propanoic acid, 2-methyl-, 2-ethyl-3-hydroxyhexyl ester, isoprene and nonanal. A comprehensive list of VOCs characterizing daily variation in room air composition can be found in Supplementary Table 2.

### Room air, but not breath, VOC profiles differ across sampling locations

**Figure 3:**
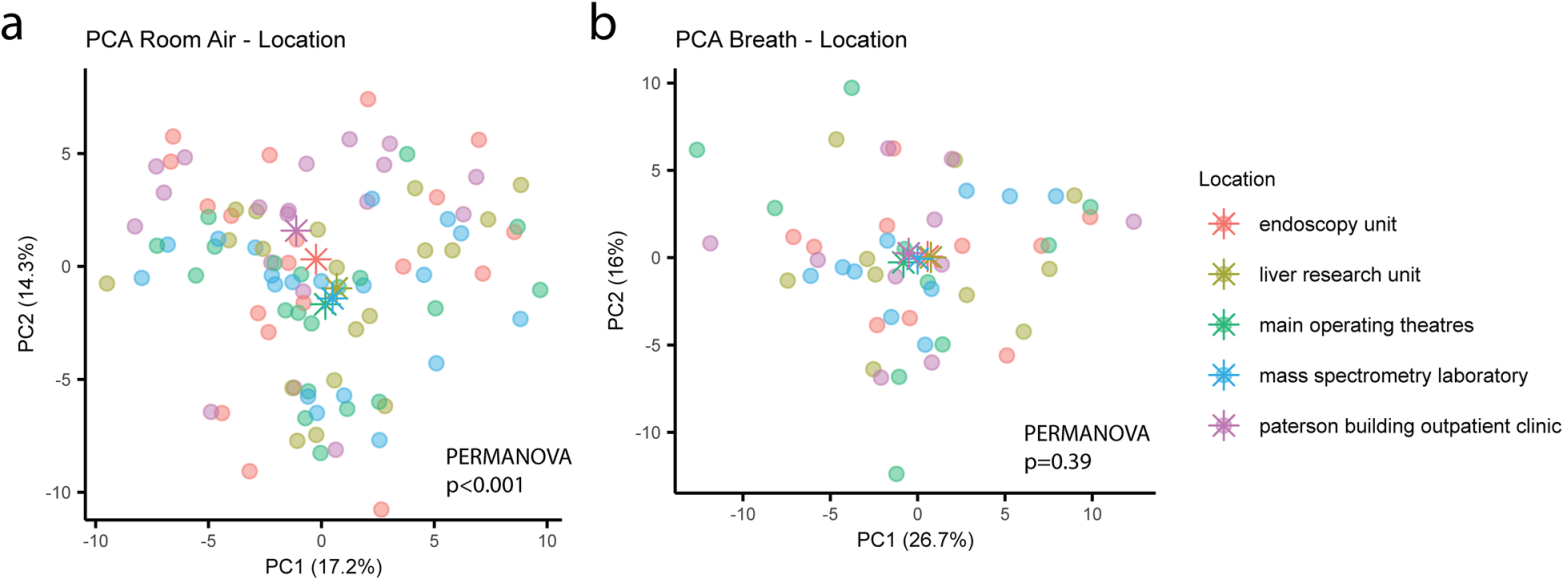
Variation of ambient room air, but not breath, VOC profiles differ across sampling locations, unsupervised analysis with PCA revealed separation between room air samples collected in different locations but did not show separation for their corresponding breath samples. Asterisks represent group centroids.

Samples were collected across five different locations: endoscopy unit, clinical research bay, operating theatre complex, outpatient clinic and a mass spectrometry laboratory within St Mary’s Hospital, London. These locations are all commonly used for patient recruitment and breath collection by our research group. Room air, as previously mentioned, was collected both in the morning and afternoon, while breath samples were only collected in the morning. PCA highlighted a separation of room air samples by location through permutational multivariate analysis of variance (PERMANOVA, R^2^ = 0.16, p < 0.001) (Fig. 3a). Thus, pairwise PLS-DA models were generated, comparing each location against all the others to identify characteristic signatures. All models were significant and VOCs with VIP score > 1 were extracted with respective loading to identify group contribution. Our results indicate that the composition of ambient air changed by location, and we identified location characteristic signature through model consensus. The endoscopy unit was characterized by higher presence of undecane, dodecane, benzonitrile and benzaldehyde. The clinical research bay (also identified as liver research unit) samples displayed more α-pinene, di-isopropyl phthalate and 3-carene. The operating theatre complex air was distinguished by a more abundant presence of branched decane, branched dodecane, branched tridecane, propanoic acid, 2-methyl-, 2-ethyl-3-hydroxyhexyl ester, toluene and 2-butenal. The outpatient clinic (Paterson building) was marked by higher levels of 1-nonanol, vinyl lauryl ether, benzyl alcohol, ethanol, 2-phenoxy-, naphthalene, 2-methoxy-, isobutyl salicylate, tridecane, and branched tridecane. Finally, the room air collected in the mass spectrometry laboratory presented more acetamide‚ 2‚2‚2-trifluoro-N-methyl-, pyridine, furan‚ 2-pentyl-, branched undecane, ethylbenzene, m-xylene, o-xylene, furfural, and ethyl anisate. Varying levels of 3-carene were present in all five locations, suggesting this VOC to be a common contaminant, with highest abundance observed in the clinical research bay. A list of consensus VOCs separating each location can be found in Supplementary Table 3. In addition, univariate analysis was performed on each VOC of interest, comparing all the locations to each other with pairwise Wilcoxon test followed by Benjamini–Hochberg correction. Boxplots for each VOC are reported in Supplementary Fig. 1. Breath VOC profiles did not appear to be affected by location as observed in PCA followed by PERMANOVA (p = 0.39) (Fig. 3b). Additionally, pairwise PLS-DA models were generated between all the different location for the breath samples too, but no significant differences were identified (p > 0.05).

## Discussion

In this study, we analysed VOC profiles within ambient room air across five commonly used locations for breath sample collection to further understand the impact of background VOCs levels on breath analysis.

Separation of room air samples across all five different locations was observed. Except for 3-carene which was present in all investigated areas, separation was driven by different VOCs, giving each location a specific signature. In the endoscopy assessment area VOCs driving separation were predominantly monoterpenes, such as β-pinene, and alkanes, such as dodecane, undecane and tridecane that are commonly found in essential oils commonly used in cleaning products^13^. Given the frequency with which the endoscopy unit is cleaned, it is likely these VOCs are a result of frequent cleaning processes within this space. In the clinical research bay, as with endoscopy, separation was predominantly due to monoterpenes, such as α-pinene, also most likely originating from cleaning products. In the operating theatres complex, the VOC signature predominantly consisted of branched alkanes. These compounds may originate from surgical instruments since they are abundant in oils and lubricants^14^. In the surgical outpatient clinic, characteristic VOCs included a selection of alcohols: 1-nonanol, found in plant oil and consequently cleaning products, and benzyl alcohol, which can be found in fragrances and local anaesthetics^15–18^. VOCs within the mass spectrometry laboratory were largely different to the other areas which was to be expected given that this was the only non-clinical area that was assessed. While some monoterpenes were present, a more homogenous group of compounds separated this area from the others (2‚2‚2-trifluoro-N-methyl-acetamide‚ pyridine, branched undecane, 2-pentyl-furan‚ ethylbenzene, furfural, ethyl anisate, o-Xylene, m-Xylene, isopropyl alcohol, and 3-Carene), including aromatic hydrocarbons and alcohols. Some of these VOCs may be secondary to chemicals used within the laboratory, consisting of seven mass spectrometry systems operating both in TD and liquid injection modes.

Strong separation of room air and breath samples was observed through PLS-DA, driven by 62 of the 113 detected VOCs. Within room air, these VOCs were exogenous and included di-isopropyl phthalate, benzophenone, acetophenone and benzyl alcohol, which are all commonly used within plasticisers and fragrances^19–22^, the latter of which can be found in cleaning products^16^. The identified chemicals in breath were a mixture of endogenous and exogenous VOCs. Endogenous VOCs largely consisted of branched alkanes which are an established by-product of lipid peroxidation^23^ and isoprene, a by-product of cholesterol synthesis^24^. Exogenous VOCs included monoterpenes such as β-pinene and D-limonene, which can be traced back to essential oils from citrus fruit (also commonly used in cleaning products) and food preservatives^13,25^. 1-Propanol can be both endogenous, deriving from amino acid breakdown, and exogenous, present in disinfectants^26^. Of the VOCs which were found in higher levels in room air compared to breath, several have been suggested as possible disease biomarkers. Ethylbenzene has been shown to be a potential biomarker for several respiratory conditions including lung cancer, COPD^27^ and pulmonary fibrosis^28^. N-Dodecane and Xylene have also been shown to be higher in patients with lung cancer compared to those without^29^ and m-cymene has been found to be higher in patients with active ulcerative colitis^30^. Therefore, even if room air differences don’t appear to affect the overall breath profiles, they might influence the levels of specific VOCs of interest, concluding that background room air monitoring is might still be essential.

Separation between room air samples collected in the morning and afternoon was also observed. Morning samples were mainly characterised by branched alkanes, which are commonly found exogenously in cleaning products and waxes^31^. The four clinical areas included within this study were all cleaned prior to the sampling of the room air which would account for this. The clinical areas were all separated by different VOCs thus this separation cannot be attributed to cleaning. Afternoon samples typically presented mixture of alcohols, hydrocarbons, esters, ketones, and aldehydes in higher levels compared to the morning samples. 1-Propanol and phenol can both be found in disinfectants^26,32^, which is expected given the regular cleaning that goes on throughout clinical areas during the day. Breath was only collected in the morning. This is due to the multiple other factors that can influence VOC level within breath over the course of the day which could not be controlled for. This includes drink and food consumption prior to breath sampling^33,34^ and different levels of exercise^35,36^.

Analysis of VOCs remains an evolving frontier in the development of non-invasive diagnostics. Standardisation of sampling remains an issue however our analysis reassuringly demonstrates no significant difference between breath samples collected at different locations. Within this study we have demonstrated that VOCs within ambient room air varies between location and time of day. However, our results also demonstrate that this does not significantly alter the profile of VOCs within exhaled breath suggesting breath sampling can be performed across varying locations without significantly impacting on results. The inclusion of multiple locations over a longer period of time and duplicate sample collection was prioritised. Finally, the separation of room air from different locations and the lack of separation in breath clearly suggests that sampling location does not significantly impact upon the composition of human breath. This is reassuring for breath analysis studies as it removes one potential confounder for the standardisation of breath collection. While having all breath samples from a single subject is a limitation of our study, it has the potential to reduce variance from other confounding factors influenced by human behaviour. Single subject study design has been successfully used previously in several studies^37^. However, further analyses are required to draw definitive conclusions. Routine sampling of room air in parallel to breath sampling is still recommended, to allow exclusion of exogenous compounds and identification of specific contaminants. We would recommend exclusion of isopropyl alcohol given its prevalence within cleaning products, especially within healthcare settings. This study was limited by the number of breath samples taken in each location and further work is required with a larger number of breath samples to confirm that there is no significant impact on the composition of human breath on the background environment in which it is samples. Furthermore, relative humidity (RH) data has not been collected and while we acknowledge that differentiations in RH might influence VOC distribution, in large scale studies, the logistical challenge is substantial both control of RH and for collection of RH data.

In conclusion, our study has demonstrated that there is variation of VOCs in ambient room air across different locations and times but that this does not appear to be the case with breath samples. Due to a small sample size, definitive conclusions regarding the impact of ambient room air on breath sampling cannot be drawn and further analysis is required and thus it is recommended to sample room air in parallel to breath to allow interrogation of any potential contaminant VOCs.

## Methods

The experiment took place over 10 non-consecutive weekdays in February 2020 at St. Mary’s Hospital, London. Each day, two breath samples and four room air samples were collected in each of the five locations, resulting in a total of 300 samples. All methods were carried out in accordance with relevant guidelines and regulations. All five sampling areas were temperature controlled at 25 °C.

### Room air sampling

Five locations were selected for room air sampling: mass spectrometry instrument laboratory, surgical outpatient clinic room, operating theatres assessment area, endoscopy assessment area and clinical research bay. Each area was selected as they are regularly utilised for participant recruitment for breath analysis by our research group.

An air sampling pump from SKC Ltd. was used to draw ambient room air across Tenax TA/Carbograph inert-coated thermal desorption (TD) tubes (Markes International Ltd, Llantrisant, UK) at a rate of 250 mL/min for 2 min, loading a total of 500 mL of ambient room air on to each TD tube. The tubes were then sealed with air-tight brass caps for transportation back to the mass spectrometry laboratory. Room air was sampled from each location in sequence between 9 and 11 a.m. each day and then again between 3 and 5 p.m. Samples were collected in duplicates.

### Breath sampling

Breath samples were collected from a single subject who performed room air sampling. The breath sampling process was performed as per the protocol approved by the NHS Health Research Authority—London—Camden & Kings Cross Research Ethics Committee (reference 14/LO/1136). The investigator provided informed written consent. For standardization purposes, the investigator had nothing to eat or drink from midnight the previous evening. A custom-made, single use Nalophan™ (PET-polyethylene terephthalate) bag with a 1000 mL capacity and a polypropylene syringe acting as a sealable mouthpiece was utilised for the collection of breath as previously described by Belluomo et al*.*^2^. Nalophan has been demonstrated to be a good medium for breath storage due to its inertness and ability to provide compound stability for up to 12 h^38^. After spending a minimum of 10 min in the location, the investigator exhaled into the sample bag during normal tidal breathing. Once filled to maximum volume, the bag was sealed with the syringe plunger. As with room air sampling, within 10 min, an air sampling pump from SKC Ltd. was used to draw breath from the bag across TD tubes: a wide bore needle without a filter was attached to a TD tube via plastic tubing and with the SKC Air Pump at the other end. The bag was needled, and breath was drawn through each TD tube at a rate of 250 mL/min for 2 min, loading a total of 500 mL of breath on to each TD tube. Samples were once again collected in duplicate to minimise sampling variability. Breath was collected in the morning only.

### Sample processing

TD tubes were cleaned using a TC-20 TD tube conditioning unit (Markes International Ltd, Llantrisant, UK) for 40 min at 330 °C with a nitrogen flow of 50 mL/min. All samples were analysed within 48 h from collection using GC-TOF–MS. An Agilent Technologies 7890A GC was paired with a TD100-xr Thermal Desorption unit and a BenchTOF Select (Markes International Ltd, Llantrisant, UK) MS. TD tubes were initially pre-purged for 1 min with the flow at 50 mL/min. Primary desorption was performed at 250 °C for 5 min at 50 mL/min He flow to desorb the VOCs onto a cold trap (Material emissions, Markes International, Llantrisant, UK) at 25 °C in split mode (1:10). Cold trap (secondary) desorption was performed at 250 °C (ballistic heating at 60 °C/s) for 3 min at 5.7 mL/min He flow, with the flow path onto GC heated constantly at 200 °C. The chromatographic column was a Mega WAX-HT, (20 m × 0.18 mm × 0.18 µm, Chromalytic, Hampshire, USA.) The column flow was set at 0.7 mL/min. Oven temperature was initially set at 35 °C for 1.9 min and was increased to 240 °C (20 °C/min with 2 min hold). The MS transfer line was maintained at 260 °C, whilst ion source (70 eV electron impact) was at 260 °C. MS analyser was set to acquire over the range of 30 to 597 m/z. Cold trap desorption (no TD tube) and conditioned, clean TD tube desorption were included in the beginning and in the end of every analytical run to ensure the absence of carryover effects. Same blank analyses had been performed right before and right after breath sample desorption to ensure that samples can be analysed sequentially without need for TD conditioning.

Following visual inspection of the chromatograms, the raw data files were analysed using Chromspace^®^ (Sepsolve Analytical Ltd.). Compounds of interested were identified from representative samples of breath and room air. Annotations were performed using NIST 2017 Mass Spectral Library based on VOC mass spectra and retention indices. Retention indices were calculated by analysing an alkane mixture (nC_8_-nC_40_, 500 μg/mL in dichloromethane, Merck, USA) 1 μL spiked onto three conditioned TD tubes via a calibration solution loading rig and analysed under the same TD-GC–MS conditions and from the raw compound list, only those with a reverse match factor > 800 were kept for analysis. Oxygen, argon, carbon dioxide and siloxanes were also removed. Finally, any compounds with a signal to noise ratio < 3 were also excluded. The relative abundance of each compound was then extracted from all data files using the compound list generated. 117 compounds were identified in breath samples versus NIST 2017. Peak picking was performed using MATLAB R2018b (Version 9.5) and Gavin Beta 3.0 software. Following further interrogation of the data with visual inspection of the chromatograms, a further 4 compounds were excluded leaving 113 compounds included in the downstream analysis. The abundance of these compounds was extracted from all 294 samples that were successfully processed. Six samples were removed due to poor data quality (leaked TD tubes). In the remained dataset, 1-tailed Pearson correlation was calculated between the 113 VOCs in the repeated measurement samples to assess reproducibility. Correlation coefficients were 0.990 ± 0.016 and p-values 2.00 × 10^–46^ ± 2.41 × 10^–45^ (arithmetic mean ± standard deviation).

### Statistical analysis

All statistical analyses were performed on R version 4.0.2 (R Foundation for Statistical Computing, Vienna, Austria). Data and code used for the analysis and to generate figures is publicly available on GitHub (https://github.com/simonezuffa/Manuscript_Breath). Integrated peaks were first log transformed and then normalised using total area normalisation. Samples for which repeated measurements were available were collapsed to the mean. The ‘ropls’ and ‘mixOmics’ packages were used to generate the unsupervised PCA models and supervised PLS-DA models. PCA allowed for the identification of 9 sample outliers. One breath sample clustered with the room air samples and therefore was felt to represent an empty tube secondary to sampling error. The other 8 samples were room air samples driven by 1,1′-biphenyl, 3-methyl. On further inspection, it was identified that all 8 samples had significantly lower VOC yields compared to the other samples, suggesting these outliers were due to manual errors in loading the tubes. Separation due to location was tested in the PCA using PERMANOVA from the ‘vegan’ package. PERMANOVA allows the identification of group separation based on centroids. This technique has been previously used in similar metabolomic studies^39–41^. The ‘ropls’ package was used to evaluate PLS-DA models significance using a randomised sevenfold cross validation and 999 permutations. Compounds with a variable importance projection (VIP) score > 1 were considered relevant for the classification and retained as significant. Loadings from the PLS-DA models were also extracted to identify group contribution. Location specific VOCs were identified through consensus of pairwise PLS-DA models. To do so, all locations VOCs profiles were tested against each other and if a VOC with VIP > 1 was constantly significant in the models and attributed to the same location, it was then considered location specific. Comparison between breath and room air samples was investigated only on samples collected during the morning since no breath sample was collected in the afternoon. Wilcoxon test was used for univariate analysis and false discovery rate was accounted applying Benjamini–Hochberg correction.

## Supplementary Information

Supplementary Information.

## Data Availability

The datasets generated during and analysed during the current study are available from the corresponding author on reasonable request.
