# Supplementary material for: Variation of volatile organic compound levels within ambient room air and its impact upon the standardisation of breath sampling
Source: Sci Rep. 2022 Sep 23;12:15887. doi: 10.1038/s41598-022-20365-7 (PMC9508138; doi:10.1038/s41598-022-20365-7)
Supplement: Supplementary file 1 — Supplementary Information. [file 41598_2022_20365_MOESM1_ESM.docx]

**Supplementary Material**

Supplementary Table 1. VOCs: Room Air vs Breath

| **Room**  **Air** | **Compound** | **CAS ID** | **Class** | **VIP Score** | **Potential Sources** |
| --- | --- | --- | --- | --- | --- |
|  | Di-isopropyl phthalate | 605-45-8 | Phthalate Ester | 1.34 | Plasticiser [1], Fragrance [2] |
|  | Benzyl alcohol | 100-51-6 | Alcohol | 1.32 | Fragrance, Solvent [3], Soap, Local Anaesthetic [4], Food (Apple, Apricot) [5] |
|  | Benzophenone | 119-61-9 | Ketone | 1.31 | Plasticiser, Fragrance [6] |
|  | Acetophenone | 98-86-2 | Ketone | 1.29 | Solvent, Plasticiser, Fragrance [7], Food (Beef, Plum, Raspberry) [8] |
|  | Azulene | 275-51-4 | Aromatic Hydrocarbon | 1.28 | Emollient, Fragrance, Skin Conditioner [9] |
|  | Naphthalene‚ 1-methyl | 90-12-0 | Aromatic Hydrocarbon | 1.27 | Fragrance, Antimicrobial [10], Food (Apple, Grape, Strawberry) [11] |
|  | Benzaldehyde | 100-52-7 | Aldehyde | 1.27 | Fragrance, Solvent, Plastic Additive [12], Food (Apple, Apricot, Cinnamon) [13] |
|  | Propanoic acid, 2-methyl-, 2-ethyl-3-hydroxyhexyl ester | 74367-31-0 | Ester | 1.26 | Food (Apricot, Plumcot) [14] |
|  | Diphenylacetylene | 501-65-5 | Alkyne | 1.25 | Fragrance [15] |
|  | Isobutyl salicylate | 87-19-4 | Salicylate | 1.24 | Fragrance, Preservative, Antimicrobial [16] |
|  | Naphthalene‚ 2-methyl- | 91-57-6 | Aromatic Hydrocarbon | 1.24 | Cigarette Smoke [17] |
|  | Benzothiazole | 95-16-9 | Heterocyclic Compound | 1.22 | Fragrance [18] , Food (Asparagus, Cocoa, Mango) [19] |
|  | Ethylbenzene | 100-41-4 | Aromatic Hydrocarbon | 1.21 | Petroleum, Inks, Pesticides, Paint [20], Food (Apricot, Cherry, Peach, Kiwi) [21] |
|  | Furfural | 98-01-1 | Aldehyde | 1.20 | Tea, Coffee, Fruits, Wholegrain Bread [22] |
|  | m-cymene | 535-77-3 | Aromatic Hydrocarbon | 1.19 | Fragrance, Skin Conditioner [23] |
|  | m-Xylene | 108-38-3 | Aromatic Hydrocarbon | 1.190 | Solvent [24], Fruit (Kiwi, Apricot) [25] |
|  | 1-Dodecanol | 112-53-8 | Alcohol | 1.19 | Fragrance [26], Toothpaste, Detergent [27] |
|  | p-Xylene | 106-42-3 | Aromatic Hydrocarbon | 1.19 | Solvent [28] |
|  | 1H-Indene‚ 2‚3-dihydro-1‚1‚3-trimethyl-3-phenyl- | 3910-35-8 | Hydrocarbon | 1.19 | Adhesive [29] |
|  | 5‚9-Undecadien-2-one‚ 6‚10-dimethyl-‚ (E)- | 3796-70-1 | Ketone | 1.18 | Cigarettes, Flavouring [30], Fragrance, Fruit [31] |
| **Breath** | Isoprene | 78-79-5 | Hydrocarbon | 1.40 | Cholesterol Biosynthesis [32] |
|  | Branched Tridecane D |  | Alkane | 1.38 | Lipid Peroxidation [33] |
|  | Branched Undecane C |  | Alkane | 1.36 | Lipid Peroxidation [33] |
|  | Branched Tridecane E |  | Alkane | 1.35 | Lipid Peroxidation [33] |
|  | Branched Tridecane C |  | Alkane | 1.32 | Lipid Peroxidation [33] |
|  | Branched Dodecane C |  | Alkane | 1.30 | Lipid Peroxidation [33] |
|  | Oxalic acid | 144-62-7 | Fatty Acid | 1.30 | Dehydroascorbic acid and glyoxylate metabolism [34] |
|  | Propane‚ 1-(methylthio)- | 3877-15-4 | Sulphur Compound | 1.29 | Kohlrabi [35] |
|  | Branched Dodecane D |  | Alkane | 1.28 | Lipid Peroxidation [33] |
|  | Branched Tridecane A |  | Alkane | 1.28 | Lipid Peroxidation [33] |
|  | Sulfide, allyl methyl | 10152-76-8 | Sulphur Compound | 1.27 | Garlic [36] |
|  | 1-Propanol | 71-23-8 | Alcohol | 1.25 | Acetone reduction, amino acid breakdown, disinfectants [37] |
|  | Branched tridecane I |  | Alkane | 1.24 | Lipid Peroxidation [33] |
|  | 2-Butenal | 4170-30-3 | Aldehyde | 1.23 | Lipid Peroxidation [38] |
|  | Branched tridecane H |  | Alkane | 1.21 | Lipid Peroxidation [33] |
|  | Branched undecane A |  | Alkane | 1.20 | Lipid Peroxidation [33] |
|  | 1-heptanol, 2-propyl | 10042-59-8 | Alcohol | 1.18 | Soap, Detergent [39] |
|  | β-pinene | 127-91-3 | Monoterpene | 1.15 | Plant Metabolite, Pine Trees, Essential Oils [40] |
|  | D-Limonene | 5989-27-5 | Monoterpene | 1.14 | Citrus Oils [41] |
|  | 1-Hexanol, 5-methyl-2-(1-methylethyl)- | 2051-33-4 | Monoterpene | 1.13 | Air Freshener [42] |

Supplementary Table 2. VOCs - AM vs PM

| **AM** | **Compound** | **CAS Registry ID** | **Class** | **VIP Score** | **Possible Sources** |
| --- | --- | --- | --- | --- | --- |
|  | Branched tridecane F |  | Alkane | 2.97 | Lipid Peroxidation [33], Cleaning products and waxes [43] |
|  | Branched hexadecane A |  | Alkane | 1.57 | Lipid Peroxidation [33], Cleaning products and waxes [43] |
|  | Branched pentadecane |  | Alkane | 1.57 | Lipid Peroxidation [33], Cleaning products and waxes [43] |
|  | Branched tetradecane A |  | Alkane | 1.49 | Lipid Peroxidation [33], Cleaning products and waxes [43] |
|  | Oxalic acid | 144-62-7 | Fatty Acid | 1.45 | Dehydroascorbic acid and glyoxylate [34] |
|  | Branched tridecane H |  | Alkane | 1.40 | Lipid Peroxidation [33], Cleaning products and waxes [43] |
|  | Branched hexadecane B |  | Alkane | 1.38 | Lipid Peroxidation [33], Cleaning products and waxes [43] |
|  | Hexacosane | 630-01-3 | Alkane | 1.37 | Flowers, Coffee [44] |
|  | Branched tridecane I |  | Alkane | 1.32 | Lipid Peroxidation [33] |
|  | Branched Tridecane A |  | Alkane | 1.28 | Lipid Peroxidation [33] |
|  | Pentacosane | 629-99-2 | Alkane | 1.26 | Pesticide [45], Avocado, Coffee [46] |
|  | Heptacosane | 593-49-7 | Alkane | 1.19 | Coffee, Avocado [47] |
|  | Branched dodecane C |  | Alkane | 1.19 | Lipid Peroxidation [33], Cleaning products and waxes [43] |
|  | Branched tridecane G |  | Alkane | 1.17 | Lipid Peroxidation [33], Cleaning products and waxes [43] |
|  | Branched Tridecane B |  | Alkane | 1.13 | Lipid Peroxidation [33], Cleaning products and waxes [43] |
|  | Branched Undecane C |  | Alkane | 1.13 | Lipid Peroxidation [33], Cleaning products and waxes [43] |
|  | n-pentadecane | 629-62-9 | Alkane | 1.12 | Pesticides [48] , Plants, Food (Egg, Mango, Mandarin, Papaya) [49] |
|  | Tetracosane | 646-31-1 | Alkane | 1.07 | Coffee, Mustard, Rose, Coconut [50] |
|  | 1-Pentadecene | 13360-61-7 | Alkene | 1.03 | Soap, Polishing Agents [51] |
| **PM** | 1-Propanol | 71-23-8 | Alcohol | 1.99 | Acetone reduction, amino acid breakdown, disinfectants [37] |
|  | Phenol | 108-95-2 | Aromatic Hydrocaron | 1.82 | Disinfectant [52] |
|  | Propanoic acid, 2-methyl-, 2-ethyl-3-hydroxyhexyl ester | 74367-31-0 | Ester | 1.66 | Plumcot, Apricot [53] |
|  | Isoprene | 78-79-5 | Hydrocarbon | 1.52 | Cholesterol Biosynthesis [54] , Rubber [55] |
|  | Nonanal | 124-19-6 | Aldehyde | 1.51 | Essential Oils, Flavouring [56] |
|  | 5‚9-Undecadien-2-one‚ 6‚10-dimethyl-‚ (E)- | 3796-70-1 | Ketone | 1.50 | Cigarettes, Flavouring [30], Fragrance, Fruit [31] |
|  | Acetophenone | 98-86-2 | Ketone | 1.35 | Solvent, Plasticiser, Fragrance [7], Food (Beef, Plum, Raspberry) [8] |
|  | Decanal | 112-31-2 | Aldehyde | 1.32 | Synthetic Citrus Oils, Fragrance, Air Freshener [57] |
|  | Benzaldehyde‚ 3‚4-dimethyl- | 5973-71-7 | Aldehyde | 1.32 | Wine [58] |
|  | 3‚4-Difluorobenzaldehyde | 34036-07-2 | Aldehyde | 1.30 | Curcumin [59] |
|  | Benzaldehyde | 100-52-7 | Aldehyde | 1.29 | Fragrance, Solvent, Plastic Additive [12], Food (Apple, Apricot, Cinnamon) [13] |
|  | D-Limonene | 5989-27-5 | Monoterpene | 1.26 | Citrus Oils [41] |
|  | Isopropyl Alcohol (2-propanol) | 67-63-0 | Alcohol | 1.260 | Antimicrobial, Solvent [60] |
|  | 1-Tetradecanol | 112-72-1 | Alcohol | 1.25 | Cosmetics [61] |
|  | Acetone | 67-64-1 | Ketone | 1.21 | Fat Metabolism, Solvent [62] |
|  | 2-pentanone | 107-87-9 | Ketone | 1.20 | Food Additive [63] |
|  | Benzophenone | 119-61-9 | Ketone | 1.16 | Plasticiser, Fragrance [6] |
|  | Benzyl alcohol | 100-51-6 | Aromatic Alcohol | 1.16 | Fragrance, Solvent [3], Soap, Local Anaesthetic [4], Food (Apple, Apricot) [5] |
|  | o-cymene | 527-84-4 | Hydrocarbon | 1.14 | Plant Oil (Seasoning, Flavouring) [64] |
|  | Furan‚ 2-pentyl- | 3777-69-3 | Furan | 1.09 | Heat-processed food and drink, human urinary metabolite, flavouring agent [65] |

Supplementary Table 3. VOCs - Locations

| **Endoscopy Unit** | **Compound** | **CAS Registry ID** | **Class** | **VIP** | **Possible Sources** |
| --- | --- | --- | --- | --- | --- |
|  | 3-Carene | 13466-78-9 | Monoterpene | 3.21 | Plant / Citrus Oils [66] |
|  | β-pinene | 127-91-3 | Monoterpene | 1.81 | Plant Metabolite, Pine Trees, Essential Oils [40] |
|  | Dodecane | 112-40-3 | Alkane | 1.66 | Essential Oils [67] |
|  | Benzonitrile | 100-47-0 | Nitrile | 1.62 | Specialty Solvent [68] |
|  | Undecane | 1120-21-4 | Alkane | 1.54 | Essential Oils [69] |
|  | 5-Hepten-2-one‚ 6-methyl- | 110-93-0 | Ketone | 1.52 | Citronella Oil, Fragrances, Flavouring [70] |
|  | Benzaldehyde | 100-52-7 | Aldehyde | 1.35 | Fragrance, Solvent, Plastic Additive [12], Food (Apple, Apricot, Cinnamon) [13] |
|  | 5‚9-Undecadien-2-one‚ 6‚10-dimethyl-‚ (E)- | 3796-70-1 | Ketone | 1.34 | Cigarettes, Flavouring [30], Fragrance, Fruit [31] |
|  | Tridecane | 629-50-5 | Alkane | 1.11 | Essential Oils [71] |
|  | Branched tridecane J |  | Alkane | 1.11 | Lipid Peroxidation [33], Cleaning products and waxes [43] |
| **Research Bay** | 3-Carene | 13466-78-9 | Monoterpene | 3.21 | Plant / Citrus Oils [66] |
|  | α-Pinene | 80-56-8 | Monoterpene | 3.1 | Essential Oils, Solvent [72] |
|  | Di-isopropyl phthalate | 605-45-8 | Phthalate Ester | 1.8 | Plasticiser [1], Fragrance [2] |
|  | Branched Undecane C |  |  | 1.25 | Lipid Peroxidation [33], Cleaning products and waxes [43] |
| **Main Theatres** | Branched decane |  | Alkane | 2.78 | Lipid Peroxidation [33], Cleaning products and waxes [43] |
|  | Propanoic acid, 2-methyl-, 2-ethyl-3-hydroxyhexyl ester | 74367-31-0 | Ester | 2.64 | Food (Apricot, Plumcot) [14] |
|  | Toluene | 108-88-3 | Monoterpene | 2.41 | Paint, Inks, Lacquer, Fuel [73] |
|  | 3-Carene | 13466-78-9 | Monoterpene | 2.37 | Plant / Citrus Oils [66] |
|  | Branched Dodecane D |  | Alkane | 2.36 | Lipid Peroxidation [33] |
|  | 2-Butenal | 4170-30-3 | Aldehyde | 1.65 | Sorbic Acid (preservative) [74] |
|  | Branched Tridecane D |  | Alkane | 1.55 | Lipid Peroxidation [33], Cleaning products and waxes [43] |
| **Laboratory** | Acetamide‚ 2‚2‚2-trifluoro-N-methyl- | 815-06-5 | Amide | 2.87 | Antimicrobial [75] |
|  | Pyridine | 110-86-1 | Heterocyclic Compound | 2.71 | Medicines, vitamins, food flavourings, paints, dyes [76] |
|  | Branched undecane A |  | Alkane | 2.5 | Lipid Peroxidation [33], Cleaning products and waxes [43] |
|  | Furan‚ 2-pentyl- | 3777-69-3 | Furan | 1.49 | Heat-processed food and drink, human urinary metabolite, flavouring agent [65] |
|  | Ethylbenzene | 100-41-4 | Aromatic Hydrocarbon | 1.42 | Petroleum, Inks, Pesticides, Paint [20], Food (Apricot, Cherry, Peach, Kiwi) [21] |
|  | Furfural | 98-01-1 | Aldehyde | 1.24 | Tea, Coffee, Fruits, Wholegrain Bread [22] |
|  | Ethyl anisate | 94-30-4 | Ester | 1.21 | Food Additive, Fragrance [77] |
|  | o-Xylene | 95-47-6 | Aromatic Hydrocarbon | 1.21 | Solvent [78] |
|  | Isopropyl Alcohol (2-propanol) | 67-63-0 | Alcohol | 1.18 | Antimicrobial, Solvent [60] |
|  | 3-Carene | 13466-78-9 | Monoterpene | 1.17 | Plant / Citrus Oils [66] |
|  | m-Xylene | 108-38-3 | Aromatic Hydrocarbon | 1.13 | Solvent [24], Fruit (Kiwi, Apricot) [25] |
| **Surgical Outpatient** | 1-Nonanol | 143-08-8 | Alcohol | 2.75 | Plant Oil [79] |
|  | Vinyl lauryl ether | 765-14-0 | Alkane | 2.61 | Coating, Chemical Intermediate [80] |
|  | 3-Carene | 13466-78-9 | Monoterpene | 2.28 | Plant / Citrus Oils [66] |
|  | Benzyl alcohol | 100-51-6 | Aromatic Alcohol | 2.16 | Fragrance, Solvent [3], Soap, Local Anaesthetic [4], Food (Apple, Apricot) [5] |
|  | Branched tridecane J |  | Alkane | 1.42 | Lipid Peroxidation [33] |
|  | Tridecane | 629-50-5 | Alkane | 1.42 | Essential Oils [71] |
|  | Ethanol‚ 2-phenoxy- | 122-99-6 | Glycol Ether | 1.28 | Insect repellent, Antiseptic, Solvent, Preservative [81] |
|  | Isobutyl salicylate | 87-19-4 | Ester | 1.09 | Fragrance, Preservative, Antimicrobial [16] |
|  | Naphthalene‚ 2-methoxy- | 91-57-6 | Aromatic Hydrocarbon | 1.07 | Cigarette Smoke [17] |


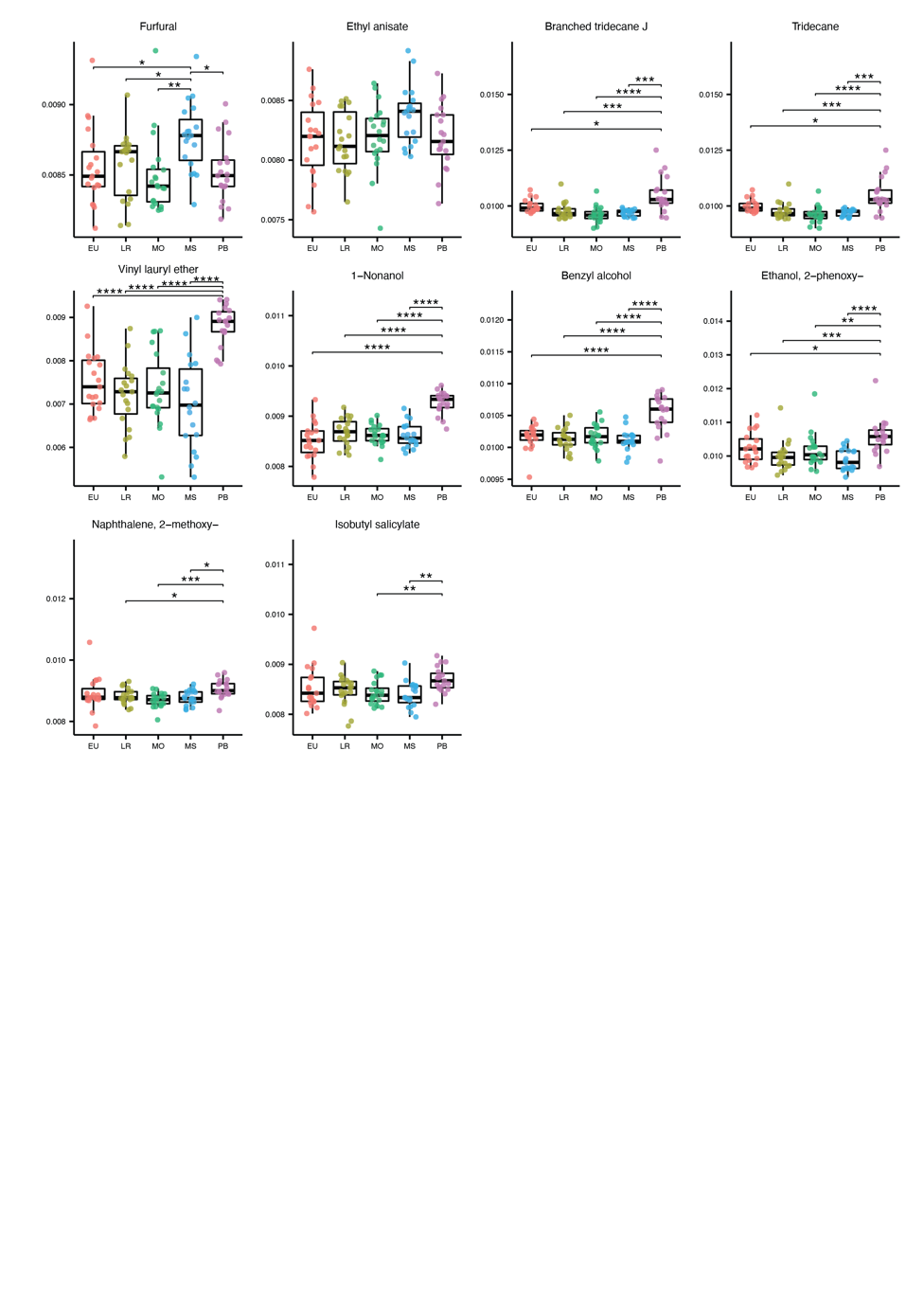

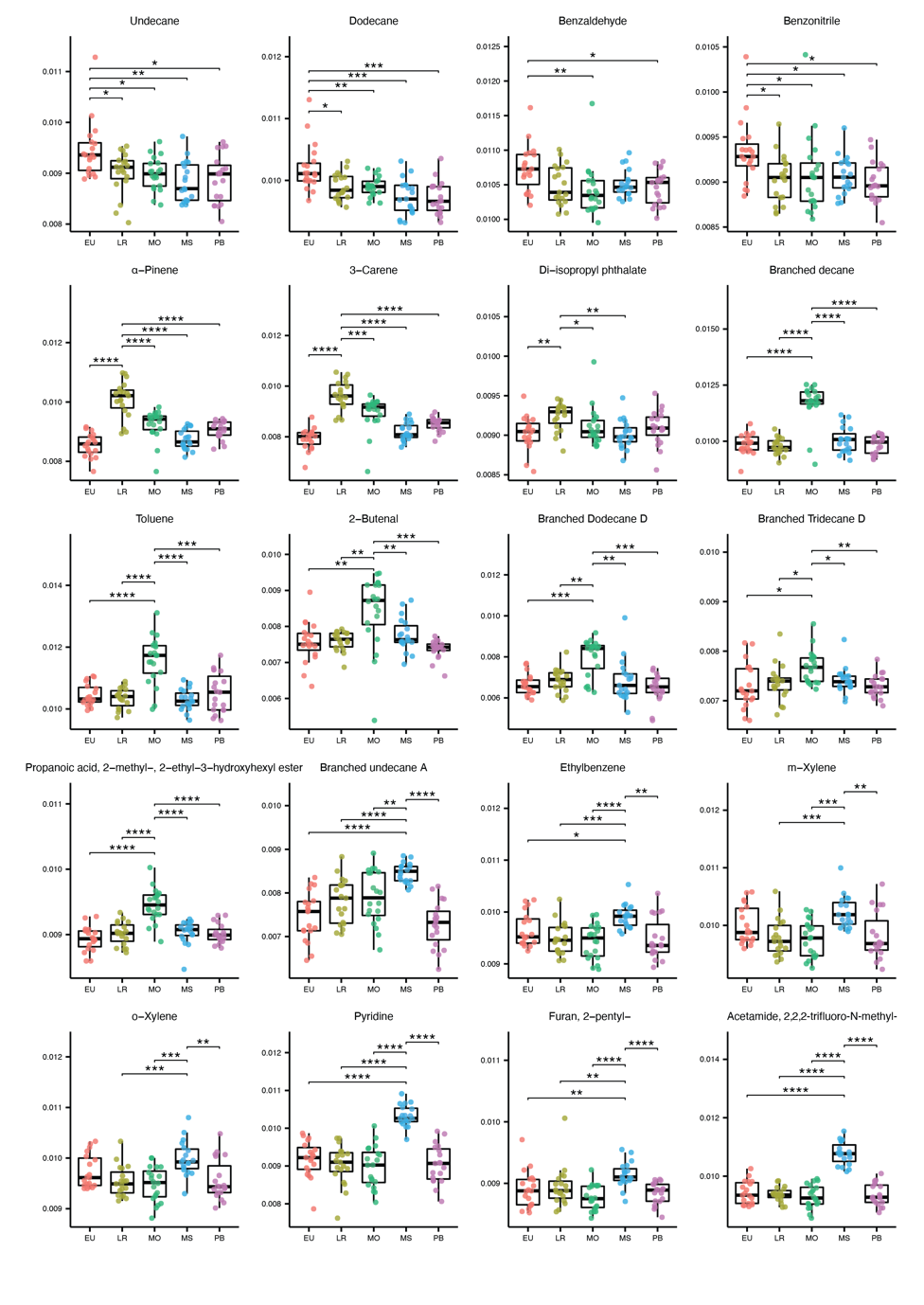
Supplementary Figure 1 – Univariate Analysis VOCs

Boxplots show first (lower) quartile, median, and third (upper) quartile. Significant was tested with pairwise Wilcoxon test followed by Benjamini-Hochberg correction. P values are represented as ∗p < 0.05, ∗∗p < 0.01, ∗∗∗p < 0.001, ∗∗∗∗p < 0.001

**References**

[1] Diisopropyl phthalate analytical standard | Sigma-Aldrich. Available at: <http://www.sigmaaldrich.com/.> Accessed Sep 22, 2021.

[2] CompTox Chemicals Dashboard - Diisopropyl phthalate. Available at: <https://comptox.epa.gov/dashboard/dsstoxdb/results?search=DTXSID2040731.> Accessed Sep 22, 2021.

[3] CompTox Chemicals Dashboard - Benzyl alcohol. Available at: <https://comptox.epa.gov/dashboard/dsstoxdb/results?search=DTXSID5020152#chemical-functional-use.> Accessed 22, September, 2021.

[4] Alfa Aesar - L03292 Benzyl alcohol, 99% Available at: <https://www.alfa.com/en/catalog/L03292/.> Accessed 22, September, 2021.

[5] The Good Scents Company - benzyl alcohol. Available at: <http://www.thegoodscentscompany.com/data/rw1001652.html.> Accessed Sep 22, 2021.

[6] Humans, IARC Working Group on the Evaluation of Carcinogenic Risks to. BENZOPHENONE. : International Agency for Research on Cancer; 2013.

[7] U.S. Environmental Protection Agency - Acetophenone. U.S. Environmental Protection Agency 1992;.

[8] The Good Scents Company - acetophenone. Available at: <http://www.thegoodscentscompany.com/data/rw1000131.html#tooccur.> Accessed Sept 22, 2021.

[9] CompTox Chemicals Dashboard - Azulene. Available at: <https://comptox.epa.gov/dashboard/dsstoxdb/results?search=DTXSID2059770#chemical-functional-use.> Accessed Sept 22, 2021.

[10] CompTox Chemicals Dashboard - 1-Methylnaphthalene. Available at: <https://comptox.epa.gov/dashboard/dsstoxdb/results?search=DTXSID9020877.> Accessed Sept 22, 2021.

[11] The Good Scents Company - 1-methyl naphthalene. Available at: <http://www.thegoodscentscompany.com/data/rw1008021.html#tooccur.> Accessed Sept 22, 2021.

[12] CompTox Chemicals Dashboard - Benzaldehyde  . Available at: <https://comptox.epa.gov/dashboard/dsstoxdb/results?search=DTXSID8039241#chemical-functional-use.> Accessed Sept 22, 2021.

[13] The Good Scents Company - Benzaldehyde . Available at: <http://www.thegoodscentscompany.com/data/rw1001492.html.> Accessed Sept 22, 2021.

[14] The Good Scents Company - Propanoic acid, 2-methyl-, 2-ethyl-3-hydroxyhexyl ester. Available at: <http://www.thegoodscentscompany.com/data/rw1656781.html.> Accessed Sept 22, 2021.

[15] CompTox Chemicals Dashboard - Benzene, 1,1'-(1,2-ethynediyl)bis-. Available at: <https://comptox.epa.gov/dashboard/dsstoxdb/results?search=DTXSID4060109#exposure.> Accessed Sept 22, 2021.

[16] CompTox Chemicals Dashboard - Isobutyl Salicylate. Available at: <https://comptox.epa.gov/dashboard/dsstoxdb/results?search=DTXSID3047186#chemical-functional-use.> Accessed Sept 25, 2021.

[17] U.S. EPA. IRIS Toxicological Review of 2-Methylnaphthalene. U.S. Environmental Protection Agency, Washington, DC, 2003 2003;.

[18] CompTox Chemicals Dashboard - Benzothiazole . Available at: <https://comptox.epa.gov/dashboard/dsstoxdb/results?search=DTXSID7024586#chemical-functional-use.> Accessed Sept 25, 2021.

[19] The Good Scents Company - Benzothiazole . Available at: <http://www.thegoodscentscompany.com/data/rw1007521.html#tooccur.> Accessed Sept 25, 2021.

[20] Ethylbenzene- ToxFAQs™. Available at: <https://www.atsdr.cdc.gov/toxfaqs/tfacts110.pdf.> Accessed Sept 25, 2021.

[21] The Good Scents Company - Ethylbenzene . Available at: <http://www.thegoodscentscompany.com/data/rw1022281.html#touses.> Accessed Sept 25, 2021.

[22] Hoydonckx HE, Rhijn WMV, Rhijn WV, Vos DED, Jacobs PA. Furfural and Derivatives. Ullmann's Encyclopedia of Industrial Chemistry: American Cancer Society; 2007.

[23] CompTox Chemicals Dashboard - m-cymene. Available at: <https://comptox.epa.gov/dashboard/dsstoxdb/results?search=DTXSID2060206#chemical-functional-use.> Accessed Sept 25, 2021.

[24] CompTox Chemicals Dashboard - m-Xylene . Available at: <https://comptox.epa.gov/dashboard/dsstoxdb/results?search=DTXSID6026298#chemical-functional-use.> Accessed Sept 25, 2021.

[25] The Good Scents Company - meta-Xylene. Available at: <http://www.thegoodscentscompany.com/data/rw1257341.html.> Accessed Sept 25, 2021.

[26] CompTox Chemicals Dashboard - 1-Dodecanol. Available at: <https://comptox.epa.gov/dashboard/dsstoxdb/results?search=DTXSID5026918.> Accessed Sept 25, 2021.

[27] 112-53-8 - 1-Dodecanol, 98% - Lauryl alcohol - Dodecyl alcohol - A12228 - Alfa Aesar. Available at: <https://www.alfa.com/en/catalog/A12228/.> Accessed Sept 25, 2021.

[28] p-Xylene. Available at: <https://pubchem.ncbi.nlm.nih.gov/compound/7809.> Accessed Sep 24, 2021.

[29] 1-Phenyl-1,3,3-trimethylindan. Available at: <https://pubchem.ncbi.nlm.nih.gov/compound/19793.> Accessed Sep 24, 2021.

[30] Geranylacetone. Available at: <https://pubchem.ncbi.nlm.nih.gov/compound/1549778.> Accessed Sep 28, 2021.

[31] Comptox Chemicals Dashboard - (E)-6,10-Dimethylundeca-5,9-dien-2-one. Available at: <https://comptox.epa.gov/dashboard/dsstoxdb/results?search=DTXSID4052053#exposure.> Accessed Sept 25, 2021.

[32] Salerno-Kennedy R, Cashman KD. Potential applications of breath isoprene as a biomarker in modern medicine: a concise overview. Wien Klin Wochenschr 2005 -03;117(5-6):180-6.

[33] Phillips M, Cataneo RN, Greenberg J, Grodman R, Gunawardena R, Naidu A. Effect of oxygen on breath markers of oxidative stress. Eur Respir J 2003 -01;21(1):48-51.

[34] Kohlmeier M. Oxalate. In: Kohlmeier M, editor. Nutrient MetabolismLondon: Academic Press; 2003, p. 232-235.

[35]  ContaminantDB - 1-(Methylthio)propane. Available at: <https://contaminantdb.ca/contaminants/CHEM033742.> Accessed Sept 25, 2021.

[36] The Good Scents Company - allyl methyl sulfide. Available at: <http://www.thegoodscentscompany.com/data/rw1038781.html#tooccur.> Accessed Sept 25, 2021.

[37] Koureas M, Kirgou P, Amoutzias G, Hadjichristodoulou C, Gourgoulianis K, Tsakalof A. Target Analysis of Volatile Organic Compounds in Exhaled Breath for Lung Cancer Discrimination from Other Pulmonary Diseases and Healthy Persons. Metabolites 2020 -8-03;10(8).

[38] Łuczaj W, Skrzydlewska E. DNA damage caused by lipid peroxidation products. Cell Mol Biol Lett 2003;8(2):391-413.

[39] 2-Propyl-1-heptanol. Available at: <https://pubchem.ncbi.nlm.nih.gov/compound/24847.> Accessed Sep 24, 2021.

[40] Salehi B, Upadhyay S, Erdogan Orhan I, Kumar Jugran A, L.D. Jayaweera S, A. Dias D, et al. Therapeutic Potential of α- and β-Pinene: A Miracle Gift of Nature. Biomolecules 2019 -11-14;9(11).

[41] D-Limonene. Available at: <https://pubchem.ncbi.nlm.nih.gov/compound/440917.> Accessed Sep 24, 2021.

[42] Steinemann A, Nematollahi N, Weinberg J, Flattery J, Goodman N, Kolev S. Volatile chemical emissions from car air fresheners. Air Quality, Atmosphere & Health 2020 November 1,;13.

[43] Maroni M, Seifert B, Lindvall T. Indoor Air Quality: A Comprehensive Reference Book. : Elsevier Science; 1995.

[44] The Good Scents Company - hexacosane. Available at: <http://www.thegoodscentscompany.com/data/rw1249621.html#toeuus.> Accessed Sept 25, 2021.

[45] Pentacosane. Available at: <https://pubchem.ncbi.nlm.nih.gov/compound/12406.> Accessed Sep 24, 2021.

[46] The Good Scents Company - pentacosane . Available at: <http://www.thegoodscentscompany.com/data/rw1272351.html#tooccur.> Accessed Sept 25, 2021.

[47] The Good Scents Company - Heptacosane. Available at: <http://www.thegoodscentscompany.com/data/rw1249251.html#tooccur.> Accessed Sept 25, 2021.

[48] Pentadecane. Available at: <https://pubchem.ncbi.nlm.nih.gov/compound/12391.> Accessed Sep 24, 2021.

[49] The Good Scents Company - pentadecane. Available at: <http://www.thegoodscentscompany.com/data/rw1272391.html.> Accessed Sept 25, 2021.

[50] The Good Scents Company - Tetracosane . Available at: <http://www.thegoodscentscompany.com/data/rw1286971.html#tooccur.> Accessed Sept 25, 2021.

[51] 1-Pentadecene. Available at: <https://pubchem.ncbi.nlm.nih.gov/compound/25913.> Accessed Sep 24, 2021.

[52] Phenol. Available at: <https://pubchem.ncbi.nlm.nih.gov/compound/996.> Accessed Sep 24, 2021.

[53] The Good Scents Company - 2-ethyl-3-hydroxyhexyl 2-methyl propanoate. Available at: <http://www.thegoodscentscompany.com/data/rw1656781.html.> Accessed Sept 25, 2021.

[54] Salerno-Kennedy R, Cashman KD. Potential applications of breath isoprene as a biomarker in modern medicine: a concise overview. Wien Klin Wochenschr 2005 -03;117(5-6):180-6.

[55] Isoprene. Available at: <https://pubchem.ncbi.nlm.nih.gov/compound/6557.> Accessed Sep 28, 2021.

[56] Nonanal. Available at: <https://pubchem.ncbi.nlm.nih.gov/compound/31289.> Accessed Sep 28, 2021.

[57] Decanal. Available at: <https://pubchem.ncbi.nlm.nih.gov/compound/8175.> Accessed Sep 28, 2021.

[58] The Good Scents Company - Benzaldehyde, 3,4-dimethyl-. Available at: <http://www.thegoodscentscompany.com/data/rw1415161.html#tooccur.> Accessed Sept 25, 2021.

[59] Chemical Book - 3,4-Difluorobenzaldehyde. Available at: <https://www.chemicalbook.com/ChemicalProductProperty_EN_CB9275911.htm.> Accessed Sept 25, 2021.

[60] Isopropyl alcohol. Available at: <https://pubchem.ncbi.nlm.nih.gov/compound/3776.> Accessed Sep 28, 2021.

[61] 112-72-1 - 1-Tetradecanol, 97+% - Myristyl alcohol - n-Tetradecyl alcohol - A19638 - Alfa Aesar. Available at: <https://www.alfa.com/en/catalog/A19638/.> Accessed Sept 25, 2021.

[62] Acetone. Available at: <https://pubchem.ncbi.nlm.nih.gov/compound/180.> Accessed Sep 28, 2021.

[63] 2-Pentanone. Available at: <https://pubchem.ncbi.nlm.nih.gov/compound/7895.> Accessed Sep 28, 2021.

[64] O-Cymene. Available at: <https://pubchem.ncbi.nlm.nih.gov/compound/10703.> Accessed Sep 28, 2021.

[65] 2-Pentylfuran. Available at: <https://pubchem.ncbi.nlm.nih.gov/compound/19602.> Accessed Sep 28, 2021.

[66] The Good Scents Company - delta-3-carene. Available at: <http://www.thegoodscentscompany.com/data/rw1014471.html.> Accessed Sept 25, 2021.

[67] Dodecane. Available at: <https://pubchem.ncbi.nlm.nih.gov/compound/8182.> Accessed Sep 28, 2021.

[68] Benzonitrile. Available at: <https://pubchem.ncbi.nlm.nih.gov/compound/7505.> Accessed Sep 28, 2021.

[69] Undecane ≥99% | Sigma-Aldrich. Available at: <http://www.sigmaaldrich.com/.> Accessed Sep 28, 2021.

[70] 6-Methyl-5-hepten-2-one. Available at: <https://pubchem.ncbi.nlm.nih.gov/compound/9862.> Accessed Sep 28, 2021.

[71] Tridecane. Available at: <https://pubchem.ncbi.nlm.nih.gov/compound/12388.> Accessed Sep 28, 2021.

[72] alpha-Pinene. Available at: <https://pubchem.ncbi.nlm.nih.gov/compound/6654.> Accessed Sep 28, 2021.

[73] Toluene. Available at: <https://pubchem.ncbi.nlm.nih.gov/compound/1140.> Accessed Sep 28, 2021.

[74] Crotonaldehyde. Available at: <https://pubchem.ncbi.nlm.nih.gov/compound/447466.> Accessed Sep 28, 2021.

[75] CompTox Chemicals Dashboard - Acetamide, 2,2,2-trifluoro-N-methyl-. Available at: <https://comptox.epa.gov/dashboard/dsstoxdb/results?search=DTXSID6061153#exposure.> Accessed Sept 25, 2021.

[76] Pyridine. Available at: <https://pubchem.ncbi.nlm.nih.gov/compound/1049.> Accessed Sep 28, 2021.

[77] Ethyl 4-methoxybenzoate. Available at: <https://pubchem.ncbi.nlm.nih.gov/compound/60979.> Accessed Sep 29, 2021.

[78] o-Xylene. Available at: <https://pubchem.ncbi.nlm.nih.gov/compound/7237.> Accessed Sep 29, 2021.

[79] 1-Nonanol. Available at: <https://pubchem.ncbi.nlm.nih.gov/compound/8914.> Accessed Sep 29, 2021.

[80] Dodecyl vinyl ether,USD,7.50/Kilogram,cas No:765-14-0,Formula:C14H28O,purity:98%min- Shanghai Jinhong Chemical Co.,Ltd. Available at: <https://www.guidechem.com/trade/dodecyl-vinyl-ether-id3448477.html.> Accessed Sep 29, 2021.

[81] 2-Phenoxyethanol. Available at: <https://pubchem.ncbi.nlm.nih.gov/compound/31236.> Accessed Sep 29, 2021.

stylefix
